# Effects of life events and stress on neutrophil functions in elderly men

**DOI:** 10.1186/1742-4933-9-13

**Published:** 2012-06-09

**Authors:** Kazumasa Tsukamoto, Kazuhiko Machida

**Affiliations:** 1 Nihon Medi-Phisics, Co., Ltd., Tokyo, 3-4-10 Shinsuna, Koto-ku, Tokyo, 136-0075, Japan; 2 Department of Preventive Medicine and Health, Welfare and Medical Policy, Graduate School of Human Sciences, Waseda University, Saitama 2-579-15, Mikajima, Tokorozawa, Saitama, 359-1192, Japan

**Keywords:** Phagocytosis, Immunosenescence, Immunity

## Abstract

**Background:**

Psychological stress or life events might significantly decrease neutrophil function in elderly individuals and lead to infectious diseases. However, relationships between these factors have not been examined in detail.

We investigated the relationships between neutrophil function measured using the nitroblue tetrazolium (NBT) reduction test and measurements of psychological stress and life events among 81 men aged over 60 years.

**Results:**

The numbers and scores for life events were significantly higher (p < 0.01, respectively) in a group with values reflecting phagocytosis below the median than in a control group.

**Conclusions:**

Chronic psychological stress due to life events decreases neutrophil functions among elderly men.

## Background

Elderly individuals tend to become more vulnerable to infections and established infections tend to become more intractable and serious with high mortality rates [1]. Decreased immune function in addition to decreased physiological function due to aging might be causes [2]. Age-related changes to the lymphocyte immune system have been documented in detail and include changes in T cell phenotypes and effector functions as well as a reduced ability of B cells to produce antibody [3].

Furthermore, dysfunction among neutrophils that play an important role in initial protection against infections [4] might also lead to susceptibility. Although the numbers of neutrophils in blood and neutrophil precursors in the bone marrow and the chemotactic responses of neutrophils reportedly remain unchanged in healthy elderly, age adversely affects human neutrophil phagocytosis and bactericidal activity against opsonized *Staphylococcus aureus* and *E. coli*[5].

On the other hand, the relationship between immune function and chronic psychological stress has been examined mainly from the viewpoint of lymphocyte function [6-8]. Some reports have indicated that chronic psychological stress influences neutrophil function [9-11]. Graham and colleagues reported that chronic psychological stress not only mimics, but also exacerbates the effects of aging on immune functions, especially lymphocyte activity [12].

Thus, chronic psychological stress or life events among elderly individuals might noticeably decrease immune function and lead to an increased risk of infectious diseases.

However, very few reports have described relationships among neutrophil functions and chronic psychological stress or life events among elderly individuals.

The present study assesses these associations using the nitroblue tetrazolium (NBT) reduction test to measure neutrophil function in whole blood against *Staphylococcus aureus*[13]. Therefore, the opsonin effect is reflected in this assay.

## Results

### Phagocytic activity relative to various parameters

We compared stress status and serum components between participants assigned to low and control groups according to median values of phagocytosis (particles/cell) (Table 1). The magnitude of chronic stress to which the participants were exposed was considered mild according to the subjective stress and stressful event scores. The group with low phagocytosis had significantly more life events and higher life event scores than the control group (1.8 ± 1.5 vs. 1.1 ± 0.8, p < 0 .01; 80.4 ± 62.1 vs. 55.5 ± 39.8, p < 0.01, respectively). The NBT reduction rate in the low phagocytosis groups was significantly lower than that of control groups (38.5 ± 11.7 vs. 51.0 ± 13.9, p < 0.05). Body mass index (BMI) and serum components did not significantly differ between the groups.

**Table 1:** Relationships between phagocytosis and other parameters

**Phagocytosis (particles/cell)**	**≥ 7.5**	**< 7.5**
N	40	41
Age	73.9 ± 5.6	71.9 ± 6.0
BMI (kg/m^2^)	23.7 ± 3.2	23.2 ± 3.8
Stress parameters		
Subjective stress score	8.9 ± 6.4	10.2 ± 6.8
Number of life events	1.1 ± 0.8	1.8 ± 1.5*
Score of life events	55.5 ± 39.8	80.4 ± 62.1*
Neutrophil functions		
Number of neutrophils	58.3 ± 7.6	60.0 ± 9.3
(× 10^2^/mm^3^)		
NBT reduction rate (%)	51.0 ± 13.9	38.5 ± 11.7^†^
Serum biochemical parameters		
TP (g/dL)	7.1 ± 0.5	6.9 ± 0.5
ALB (g/dL)	4.2 ± 0.3	4.2 ± 0.3
T-CHO (mg/dL)	173.3 ± 32.3	174.8 ± 31.1
TG (mg/dL)	120.7 ± 93.7	112.3 ± 65.7

Values are means ± SD. ^†^P < 0.05, *P < 0.01.

### Superoxide production relative to various parameters

The participants were assigned to groups according to NBT reduction rates and the parameters described above were compared (Table 2). The amount of phagocytosis was significantly lower in the group with a low NBT reduction rate than in the control group (6.4 ± 1.7 vs. 9.4 ± 3.5, p < 0.01). Although subjective stress scores, scores for life events, and the number of life events tended to be higher in the group with a low NBT reduction rate than in controls, the difference did not reach significance. Both BMI and serum components did not significantly differ between the groups.

**Table 2:** Relationships between NBT reduction rates and other parameters

**NBT reduction rate (%)**	**≥ 42**	**< 42**
N	41	40
Age	73.8 ± 5.7	71.9 ± 6.0
BMI (kg/m^2^)	23.6 ± 3.2	23.3 ± 3.8
Stress parameters		
Subjective stress score	9.1 ± 6.5	9.9 ± 6.7
Number of life events	1.3 ± 1.1	1.6 ± 1.4
Score of life events	64.3 ± 47.1	72.2 ± 60.2
Neutrophil functions		
Number of neutrophils	58.5 ± 8.4	60.3 ± 8.6
(×10^2^/mm^3^)		
Phagocytosis (particle/cell)	9.4 ± 3.5	6.4 ± 1.7*
Serum biochemical parameters		
TP (g/dL)	7.0 ± 0.6	7.0 ± 0.5
ALB (g/dL)	4.1 ± 0.3	4.2 ± 0.3
T-CHO (mg/dL)	172.3 ± 34.0	176.0 ± 28.8
TG (mg/dL)	118.2 ± 92.1	114.4 ± 65.8

Values are means ± SD. *P < 0.01.

### Stepwise multiple regression analysis

**Figure 1:**
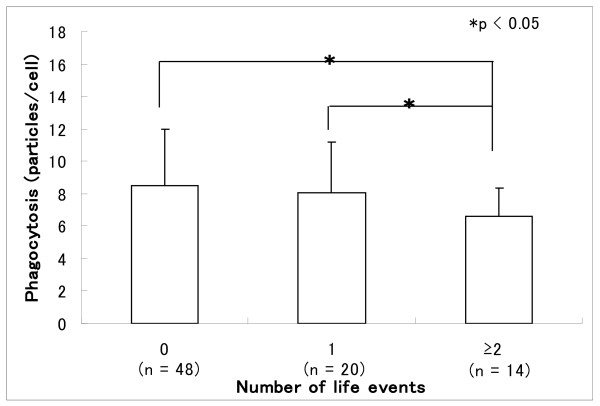
Relationship between number of life events over the past 18 months and phagocytosis by neutrophils among elderly men.

Table 3 shows the results of stepwise multiple regression analysis using neutrophil function as a dependent parameter. The NBT reduction rate was excluded from independent parameters when phagocytosis was the dependent parameter because it induced considerably more neutrophilic superoxide production. The number of life events was selected as a significant independent parameter of phagocytosis (partial regression coefficient, -0.383, p < 0.01), whereas phagocytosis was selected as significant independent parameter for the NBT reduction rate (partial regression coefficient, 0.473, p < 0.01). Figure 1 shows the relationship between the number of life events and phagocytic activity. Increasing numbers of life events tended to reduce phagocytic activity (without and with one, or two or more stress events: 8.5 ± 3.6 vs. 8.0 ±3.1 and 6.8 ± 1.4: p < 0.05 among the groups).

**Table 3:** Stepwise multiple regression analysis

	**Dependent parameters**	
	**Phagocytosis**	**NBT reduction rate**
**Independent parameters**		
Subjective stress score	NS	NS
Number of life events	−0.383*	NS
Score of life events	NS	NS
Number of neutrophils	NS	NS
Phagocytosis	-	0.473*

Values are partial correlation coefficients ; *P < 0.01. NS, not significant.

## Discussion

### Effect of chronic psychological stress on immune functions among the elderly

Graham and colleagues reported that chronic psychological stress not only mimics, but also exacerbates the effects of aging on immune functions, especially lymphocyte activity [12].

The present findings of neutrophil function coincided with these phenomena, suggesting that chronic psychological stress significantly influences neutrophil function among the elderly. These results support the hypothesis that even a relatively mild degree of chronic psychological stress can cause neutrophil dysfunction in elderly persons.

Less phagocytosis is associated with higher scores and numbers of life events (Table 1), but a higher score of life events was not related to phagocytosis in a stepwise multiple regression analysis (Table 3). This might be attributable to the fact that these two parameters closely correlate but the latter is more suitable to the regression model for explaining neutrophil phagocytic activity. Thus, the number, rather than the type of life events experienced by the elderly might be more important for neutrophil function.

We previously found better neutrophil function in the elderly with, than without stress coping factors such as hobbies, religion, having a pet or maintaining close family ties [14]. Therefore, stress coping factors might be important to maintain neutrophil functions among elderly persons who experience stressful life events.

Phagocytosis correlated with a reduction in NBT (Tables 1,3). However, the association between NBT reduction rate and the number of life events was not remarkable (Tables 2 and 3), although the group with a low NBT reduction tended to experience more life events. The results of a mouse study of chronic psychological stress (crowding) were similar in that phagocytosis was significantly reduced but the effects on NBT reduction activity were not remarkable [15]. The findings of a human study of chronic stress were similar [9]. Although careful consideration is warranted, phagocytic activity might be more sensitive to chronic stress than NBT reductions.

### Obesity and neutrophil functions

No associations between neutrophil functions and serum biochemical factors or body mass index (BMI) were identified in the present study (Tables 1 and 2). Since we previously reported that low serum total protein is associated with low neutrophil phagocytic activity in the elderly [14], we measured serum total protein and albumin levels. Obesity and related metabolic disorders affect immune functions. A recent study has found that changes in leptin and adiponectin levels accompanied by obesity are closely involved in immunosenescence [16]. Therefore, we measured serum lipid levels and the BMI of the participants. The results indicated no tendencies towards obesity or malnutrition among the elderly participants.

### Humoral factors involved in neutrophil functions

The phagocytic and sterilizing effects of neutrophils are easily affected by various humoral factors. For example, cortisol, which is elevated by stress, causes immunosenescence including neutrophil dysfunction [11,17]. Because the NBT reduction test is used to measure neutrophil function in whole blood but not isolated neutrophils, these humoral factors including opsonin effect must be reflected in test results. Dysfunction/lack of antibody against the bacteria produced by B cells might also be attributed to dysfunctional neutrophil phagocytosis among elderly individuals who have experienced life events.

### Health impact of neutrophil dysfunction

Trends between phagocytosis and NBT reduction rate coincided throughout the study (Tables 1–3). Bacterial phagocytosis induced neutrophilic superoxide production, which seems a highly purpose-adaptable phenomenon. Reduced neutrophil phagocytic activity is closely linked to decreased superoxide production, which might cause susceptibility in elderly individuals.

Neutrophil dysfunction due to aging and stress events might thus lead to the pathogenesis and progression of infection, with which chronic psychological factors have also been linked [18]. Thus the impact of neutrophil dysfunction caused by aging and chronic psychological stress on health outcomes needs to be demonstrated.

### Limitations

This study has some limitations. Firstly, elderly female individuals were not included. Secondly, we did not measure cortisol and cytokine levels. Further studies are warranted to clarify the effect of stress-induced alterations in humoral factors on neutrophil functions in the elderly.

## Conclusions

Our findings indicated that neutrophil functions are significantly suppressed in elderly individuals exposed to chronic psychological stress due to life events.

## Methods

### Study participants

Eighty-one men aged over 60 (72.8 ± 3.8) years who were members of a support group for senile individuals in Sayama city voluntarily provided written informed consent to participate in this study. The study proceeded in accordance with the ethical standards of the Declaration of Helsinki. Three men who were affected by colds or who received anti-influenza preparations on the day of survey were excluded.

### Stress survey

The degree of subjective stress was examined using the Stress Check List for Self (SCL-S) developed by Katsura et al. in which scores of ≤ 10, 11 ~ 20 and ≥ 21 indicate mild, moderate and severe stress, respectively [19].

The number of stressful life events experienced during the past 18 months and total scores for life events were assessed according to The Social Read-Adjustment Rating Scale developed by Holmes et al. [20]. Forty-one life events are interpreted by the scores in this scale such as death of a spouse, 100 (maximum); divorce, 75 and a minor violation of law, of 11 (minimum). The sum of these scores is also evaluated as health impact; scores of > 300, 150 ~ 299 and < 150 indicate high, moderate and a slight risk of illness, respectively [20].

### Serum biochemical analysis

Peripheral venous blood samples were obtained after the stress survey by antecubital venipuncture during the early morning after an overnight fast. After allowing the blood to clot at room temperature, serum samples were separated by centrifugation at 1,000 × g for 10 min and then serum total protein (TP), albumin (ALB), total cholesterol (T-CHO) and triglyceride (TG) were measured using the assay kits recommended for use with the Clinical Chemistry Analyzer GL-7000 (Shimadzu Co. Ltd., Tokyo).

### Assays of neutrophil function

The NBT reduction test [13] was performed continuously by incubating a mixture of whole blood, *Staphylococcus aureus* suspension and 0.1 % NBT (0.1 mL each) at 37°C for 20 min. Smears were immediately prepared thereafter on site and cells were visualized by May-Giemsa staining.

After the survey and the measurements, the ratio (%) of cells containing formazan crystals and the number of ingested bacteria in neutrophils were determined by microscopic examination in a blinded manner at our laboratory [21,22].

### Statistical analysis

Statistical values are expressed as means ± SD and differences in variables between two groups were compared using a t-test. Values from three groups were compared using a one way ANOVA and the Bonferroni test. Stepwise multiple regression analysis included neutrophil functions as dependent parameters to calculate partial regression coefficients. P values of < 0.05 were considered significant.

## Abbreviations

ALB, Albumin; BMI, Body mass index; NBT, Nitroblue tetrazolium; SCL-S, Stress check list for self; T-CHO, Total cholesterol; TG, Triglyceride.

## Competing interests

The authors declare that they have no competing interests.

## Authors' contributions

KT conducted the survey and had the overall responsibilities of the study design and statistical analysis, and wrote the manuscript. KM had the overall responsibilities of conducting the survey and study design. All authors have read and approved the final manuscript.

## References

[B1] Castle SC Clinical relevance of age-related immune dysfunction Clin Infect Dis 2000 31 578 585 10.1086/313947 10987724

[B2] Gomez CR Boehmer ED Kovacs EJ The aging innate immune system Curr Opin Immunol 2005 17 457 462 10.1016/j.coi.2005.07.013 16084711

[B3] Load JM Butcher S Killampali V Lascelles D Salmon M Neutrophil aging and immunesenescence Mech Aging Dev 2001 122 1521 1535 10.1016/S0047-6374(01)00285-8 11511394

[B4] Ottonello L Dapino P Pastorino G Dallegri F Sacchetti C Neutrophil dysfunction and increased susceptibility to infection Eur J Clin Invest 1995 25 687 692 10.1111/j.1365-2362.1995.tb01987.x 7498244

[B5] Simell B Vuorela A Ekström N Palmu A Reunanen A Meri S Käyhty H Väkeväinen M Aging reduces the functionality of anti-pneumococcal antibodies and the killing of Streptococcus pneumoniae by neutrophil phagocytosis Vaccine 2011 29 1929 1934 10.1016/j.vaccine.2010.12.121 21236231

[B6] Schleifer S Keller SE Cammerino M Thronton JC Stein M Suppression of lymphocyte stimulation following bereavement JAMA 1983 250 374 347 10.1001/jama.1983.03340030034024 6854901

[B7] Phillips AC Carroll D Evans P Bosch JA Clow A Hucklebridge F Der G Stressful life events are associated with low secretion rates of immunoglobulin A in saliva in the middle aged and elderly Brain Behav Immun 2006 20 191 197 10.1016/j.bbi.2005.06.006 16055305

[B8] Gerra G Monti D Panerai AE Sacerdote P Anderlini R Avanzini P Zaimovic A Brambilla F Franceschi C Long-term immune-endocrine effects of bereavement: Relationships with anxiety levels and mood Psychiatry Res 2003 121 145 158 10.1016/S0165-1781(03)00255-5 14656449

[B9] Dekaris D Sabioncello A Mazuran R Rabatić S Svoboda-Beusan I Racunica NL Tomasić J Multiple changes of immunologic parameters in prisoners of war: Assessments after release from a camp in Manjaca, Bosnia JAMA 1993 270 595 599 10.1001/jama.1993.03510050061028 8331758

[B10] Bartlett JA Demetrikopoulos MK Schleifer SJ Keller SE Phagocytosis and killing of Staphylococcus aureus: Effects of stress and depression in children Clin Diagn Lab Immunol 1997 4 362 366 9144378 10.1128/cdli.4.3.362-366.1997 PMC170533

[B11] Arranz L Guayerbas N De la Fuente M Impairment of several immune functions in anxious women J Psychosom Res 2007 62 1 8 10.1016/j.jpsychores.2006.07.030 17188114

[B12] Graham JE Christian LM Kiecolt-Glaser JK Stress, age, and immune function: Toward a lifespan approach J Behav Med 2006 29 389 400 10.1007/s10865-006-9057-4 16715331 PMC2805089

[B13] Digregorio KA Cilento EV Lantz RC Measurement of superoxide release from pulmonary alveolar macrophages Am J Physiol 1987 252 677 683 10.1152/ajpcell.1987.252.6.C677 3035935

[B14] Tsukamoto K Suzuki K Machida K Saiki C Murayama R Sugita M Relationships between lifestyle factors and neutrophil functions in the elderly J Clin Lab Anal 2002 16 266 272 10.1002/jcla.10152 12357457 PMC6808157

[B15] Tsukamoto K Machida K Ina Y Kuriyama T Suzuki K Murayama R Saiki C Effects of crowding on immune functions in mice Nippon Eiseigaku Zasshi 1994 49 827 836 10.1265/jjh.49.827 7807710

[B16] Craft MK Read MJ Immunologic changes in obesity Crit Care Clin 2010 26 629 631 10.1016/j.ccc.2010.06.007 20970048

[B17] Baltch AL Hammer MC Smith RP Bishop MB Sutphen NT Egy MA Michelsen PB Comparison of the effect of three adrenal corticosteroids on human granulocyte function against Pseudomonas aeruginosa J Trauma 1986 26 525 533 10.1097/00005373-198606000-00006 3088287

[B18] Cohen S Keynote Presentation at the Eight International Congress of Behavioral Medicine: the Pittsburgh Common Cold Studies: Psychological predictors of susceptibility to respiratory infectious illness Int J Behav Med 2005 12 123 131 10.1207/s15327558ijbm1203_1 16083315 PMC7091093

[B19] Koyama Y Machida K Katayama K Ogawa N Ikehara S Xia MQ Liu CY Machida K Relationship between lifestyle and oral health in Chinese elderly Nippon Eiseigaku Zasshi 2006 61 53 62 10.1265/jjh.61.53 16506655

[B20] Holmes TH Rahe RH The social readjustment rating scale J Psychosom Res 1967 11 213 218 10.1016/0022-3999(67)90010-4 6059863

[B21] Suzuki K Sato H Kikuchi T Abe T Nakaji S Sugawara K Totsuka M Sato K Yamaya K Capacity of circulating neutrophils to produce reactive oxygen species after exhaustive exercise J Appl Physiol 1996 81 1213 1222 8889756 10.1152/jappl.1996.81.3.1213

[B22] Kuriyama T Machida K Suzuki K Importance of correlations between phagocytic activity and superoxide production of neutrophils under conditions of voluntary exercise and stress J Clin Lab Anal 1996 10 458 464 10.1002/(SICI)1098-2825(1996)10:6<458::AID-JCLA25>3.0.CO;2-V 8951620

